# A case of uterine inclusion cysts in a sow

**DOI:** 10.1186/s40813-021-00237-8

**Published:** 2021-11-01

**Authors:** Elisa Ruiz-Riera, Miquel Nofrarias, Bernat Martí-Garcia, Mariano Domingo, Joaquim Segalés, Enric Vidal

**Affiliations:** 1 Servei de Diagnòstic de Patologia Veterinària (SDPV), Departament de Sanitat i d’Anatomia Animals, Universitat Autonòma de Barcelona, 08193 Bellaterra (Cerdanyola del Vallès), Barcelona, Spain; 2 UAB, Centre de Recerca en Sanitat Animal (CReSA, IRTA-UAB), Campus de la Universitat Autònoma de Barcelona, Barcelona, Spain; 3 OIE Collaborating Centre for the Research and Control of Emerging and Re-Emerging Swine Diseases in Europe (IRTA-CReSA), Bellaterra, Barcelona, Spain; 4 IRTA, Centre de Recerca en Sanitat Animal (CReSA, IRTA-UAB), Campus de la Universitat Autònoma de Barcelona,, 08193 Bellaterra (Cerdanyola del Vallès), Barcelona, Spain

**Keywords:** Serosal inclusion cysts, Uterus, Reproductive tract, Swine

## Abstract

**Background:**

Serosal inclusion cysts are thin walled-structures located on the peritoneal surface of the uterus, frequently observed as multiple cystic structures in aggregates or grape-like clusters containing a clear, non-viscous fluid. In human and veterinary medicine, they are thought to be developed under hormonal effects, or after manipulation or inflammation of the reproductive tract. However, they have not yet been described in swine.

**Case presentation:**

A uterus of a 3-year-old crossbreed sow was condemned at slaughter due to the presence of multiples cystic cavities attached to the serosal surface. Microscopically, multiple cystic dilations emerging from the serosa were lined by a simple and flattened epithelium (cytokeratine positive and vimentin negative on immunohistochemistry) supported by a subepithelial layer of collagen. Grossly and histologically, they were diagnosed as serosal inclusion cysts.

**Conclusion:**

To the authors’ knowledge, this report represents the first description of serosal inclusion cysts in sows. These lesions should be taken into consideration within the differential diagnostic list of cystic peritoneal lesions such as cystic neoplasms, congenital cysts, and parasitic diseases.

## Background

Serosal inclusion cysts are thin-walled structures located on the peritoneal surface of the uterus, frequently shown as multiple cystic structures in aggregates or grape-like clusters containing clear non-viscous fluid [1, 2]. In the veterinary literature there are scant references regarding this cyst type, with cases described mostly in the dog [3, 4], but also in other species such as cats or ruminants (including one publication in buffaloes) [2, 5, 6]. Cysts are attached to the anti-mesometrial side and are mainly described in the female dog during the postpartum period but have also been described in cases with pyometra. This is considered an incidental finding in this animal species [1, 7].

In human obstetrical literature they are known as peritoneal inclusion cysts because are often observed in peritoneum affecting mostly uterus, but also other abdominal organs. Although aetiology is not well understood, some studies suggest that they are developed under hormonal effects. Management options in women patients include from observation and follow-up of the patient to ovariohysterectomy [8].

This report describes, for the first time in the porcine species, the gross pathology and histology of uterine inclusion cysts in a sow.

## Case presentation

**Fig. 1:**
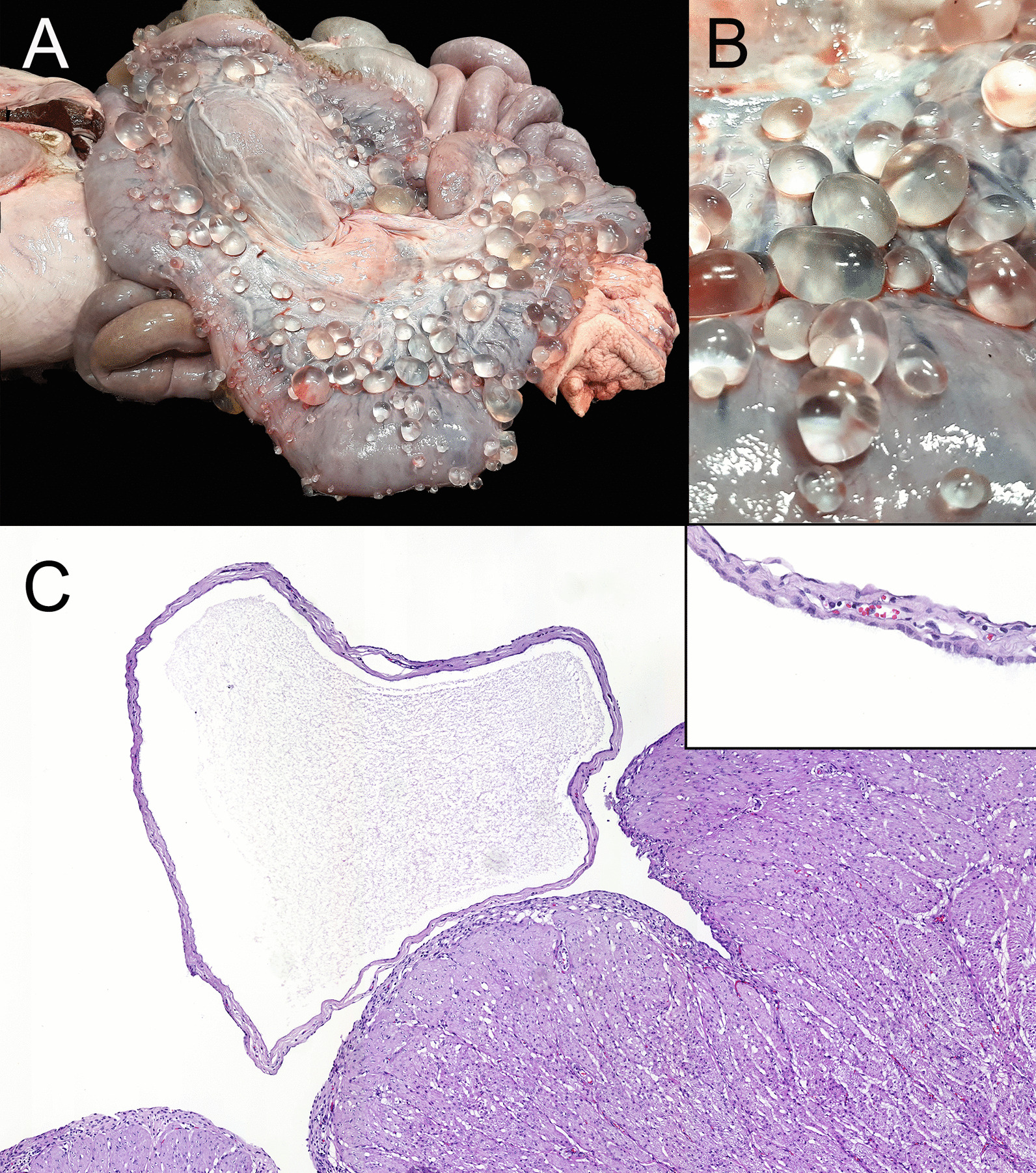
Macroscopic and microscopic findings. **a** Gross image of the sow uterus, with multiple cystic structures of variable diameters attached to the serosa. **b** Detail of the cysts. Notice the transparent wall and clear fluid that fills the cysts. **c** Histologically, cysts were lined by a simple and flattened epithelium with no atypia, with the lumen filled with hyaline proteinaceous material (corresponding to fluid). The subepithelial lamina consisted mainly of collagen fibres. Insert: detail of the cysts lining epithelium. Externally, cysts are also lined by peritoneal epithelium. H&E. × 5 and insert × 80

During routine swine post-mortem examination at slaughterhouse, a uterus of a 3-year-old crossbreed sow was condemned due to the presence of multiple cystic cavities attached to the serosal surface. Cysts were variable in diameter (from 0.5 to 5 cm) and were filled with transparent fluid (Fig. 1a,b).

Samples were submitted to the Slaughterhouse Support Network (Servei de Suport a Escorxadors [SESC] IRTA-CReSA]) [9] to rule out a presumptive diagnosis of multiple *Cysticercus tenuicollis* cysts or other potential lesions compatible with zoonotic pathogens. A container with formaldehyde (10%) was received with a 9 cm in length piece of uterus with multiple serosal cysts of 0.3 to 2 cm diameter. Sections were made and embedded in paraffin blocks. Subsequently, sections for histological examination were obtained and stained with haematoxylin and eosin. Also, immunohistochemical techniques to detect cytokeratin and vimentin intermediate filaments were performed on the studied samples to confirm or rule out the epithelial or mesenchymal origin of the cysts’ cell covering layer, respectively.

**Fig. 2:**
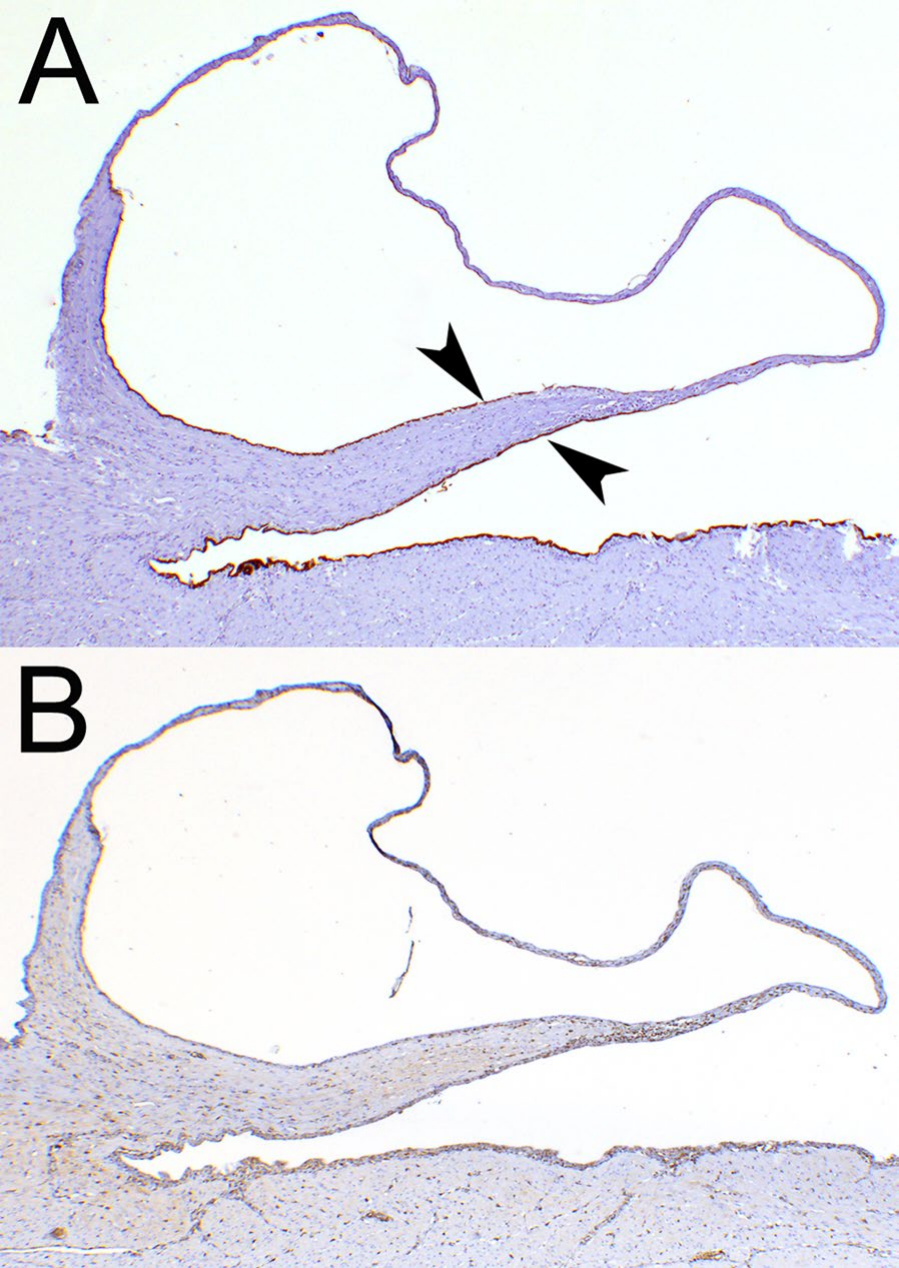
Immunohistochemical characterization. **a** Both the inner and outer epithelium (arrows) were positively stained with pan-cytokeratin antibody, the immunolabeling was more intense at the base of the cyst and more faint as the epithelium flattened. **b** The epithelium was negative to vimentin immunolabeling

Histological examination revealed multiple cystic dilations emerging from the uterus serosa that were lined by a simple and flattened epithelium, with no cellular atypia. Externally and concurrently, the cysts and the uterus were recovered by an also simple and flattened epithelium corresponding to the peritoneum. The subepithelial lamina consisted mainly of collagen fibres. In the union between the cysts and the uterus, normal smooth muscle fibres with no pathological alterations were also observed (Fig. 1c). Immunohistochemistry revealed that the epithelium on both sides of the cyst walls was positive to cytokeratin and negative to vimentin, suggesting a mesothelial origin (Fig. 2). Histological findings were consistent with serosal inclusion cysts.

## Discussion and conclusions

There are few publications regarding benign and malignant neoplastic lesions found in the reproductive tract of sows. In a recently published review from carcass inspection in domestic pigs [10], the most frequently reported lesions of the reproductive tract in the sow were leiomyoma, fibroma, cystadenoma, fibroleiomyoma and carcinoma, with occasional metastatic lymphomas described. No inclusion cysts have been reported so far in swine [10, 11].

As pigs are progressively becoming pet animals, life expectancy lengthens, with more reproductive tract disturbances reported as it happens in the feline and canine homologues [1]. In a recently published case series of pet adult female pigs [12], cystic endometrial hyperplasia was a common finding as well as uterine tumours detected during ovariohysterectomy or at necropsy. Tumours identified were leiomyoma, fibroleiomyoma and leiomyosarcoma [11].

Serosal inclusion cysts are occasionally described in the veterinary literature, mostly in dogs and cats [1, 3–5, 7]. Lesions are located on the surface of the uterus, and it is hypothesized that they develop in areas with mild peritoneal reactivity, which may cause entrapment of small areas of serosa. Subsequent eventual secretions of this serosa produce very thin-walled cysts that emerge and protrude from the surface into the peritoneal cavity. Cysts may be single or multiple, and frequently occur in clusters on any area of the peritoneum covering the uterine horns or body. They are referred to contain clear, non-viscous fluid. These lesions are commonly seen in bitches following caesarean section, where the uterus has been previously handled and manipulated or in post-partum uterine involution [1, 2].

In the human literature, multiple denominations are used for the same finding, being peritoneal inclusion cysts the most frequently used terminology, but also, they have been referred as benign (multi) cystic peritoneal mesotheliomas, inflammatory cysts of the peritoneum, postoperative peritoneal cysts, and benign papillary peritoneal cystosis. The aetiology and pathogenesis are not well understood but, in all cases described, the patients have a clinical history of women of reproductive age with abdominal or pelvic surgeries, or inflammation in the abdominal cavity or reproductive tract. Evidence suggests that hormone shifts influence the course of the disease. Management options range from observation and follow-up of the patient to surgical resection of the reproductive tract. Cure is thought to be achievable with surgical resection only [8]. No apparently vital implication occurs for women, female dogs nor cats.

Since the current case is the first description of serosal inclusion cysts in swine to the authors’ knowledge, probably it is a very uncommon incidental finding at slaughter. However, no clinical signs are expected in affected animals, since it is a non-infiltrating but a soft compressing structure into the abdomen. Therefore, it is also probable that veterinarians, at abattoir or also during a necropsy, may just overlook or minimise the importance of this finding in the eventuality of being present. On the other hand, there are other conditions implying cysts in the reproductive tract of the sow. Epithelial neoplasms of the ovary must also be considered. An ovarian papillary cystadenocarcinoma has been described with multiple clustered fluid filled cystic lesions of variable diameters throughout the abdominal serosa [10]. Another possible differential diagnosis with cystic implication in abdominal surfaces is congenital serous cysts. These latter cysts are attached to the capsule of the liver and have been reported in many different species, including swine, and are considered rare incidental findings during laparotomies or necropsies. Congenital serous cysts are usually small and multiple, but can be isolated and grow extremely large and become symptomatic [13–15]. Parasitic diseases can also cause cystic formations affecting serosal surfaces. *Cysticercus tenuicollis* cysts (*Taenia hydatigena* larval stage) are shown as transparent vesicles on the surface of the liver, omentum, or other abdominal viscera. Cysticerci are subspherical, whitish, translucent, fluid‐filled cysts about 1 cm in diameter. Visible through the surface of the cyst there is a white, 1–2 mm spot that is an inverted protoscolex that will become the scolex of the adult tapeworm.

In conclusion, this is the first case of uterine or peritoneal inclusion cysts described in a sow. Although it is not a life-threatening condition and probably very infrequent, it should be taken into consideration for the differential diagnosis in animals of reproductive age showing cysts in the peritoneum, discarding other processes such as parasitic or congenital (located in the liver) cysts or other neoplastic and hyperplastic cystic differential diagnoses.

## Data Availability

All data generated or analysed during this study are included in this published article.

## Abbreviations

SDPV: Servei de Diagnòstic de Patología Veterinària
SESC: Servei de Suport a Escorxadors
CReSA: Centre de Recerca en Sanitat Animal
UAB: Universitat Autònoma de Barcelona
IRTA: Institut de Recerca i Tecnologia Agroalimentàries
